# Reproducibility and Relative Validity of a Short Food Frequency Questionnaire in 9–10 Year-Old Children

**DOI:** 10.3390/nu8050271

**Published:** 2016-05-07

**Authors:** Pouya Saeedi, Sheila A. Skeaff, Jyh Eiin Wong, Paula M. L. Skidmore

**Affiliations:** 1 Department of Human Nutrition, University of Otago, P.O. Box 56, Dunedin 9054, New Zealand; pouya.saeedi@otago.ac.nz (P.S.); sheila.skeaff@otago.ac.nz (S.A.S.); 2 School of Healthcare Sciences, Faculty of Health Sciences, Universiti Kebangsaan Malaysia, Kuala Lumpur 50300, Malaysia; wjeiin@ukm.edu.my

**Keywords:** food frequency questionnaire, reproducibility, validity, children, New Zealand

## Abstract

The aim of this study was to assess the reproducibility and validity of a non-quantitative 28-item food frequency questionnaire (FFQ). Children aged 9–10 years (*n* = 50) from three schools in Dunedin, New Zealand, completed the FFQ twice and a four-day estimated food diary (4DEFD) over a two-week period. Intraclass correlation coefficients (ICC) and Spearman’s correlation coefficients (SCC) were used to determine reproducibility and validity of the FFQ, respectively. Weekly intakes were estimated for each food item and aggregated into 23 food items/groups. More than half of the food items/groups (52.2%) had an ICC ≥0.5. The median SCC between FFQ administrations was 0.66 (ranging from 0.40 for processed meat to 0.82 for sweets and non-dairy drinks). Cross-classification analysis between the first FFQ and 4DEFD for ranking participants into thirds showed that breakfast cereals had the highest agreement (54.0%) and pasta the lowest (34.0%). In validity analyses, 70% of food items/groups had a SCC ≥0.3. Results indicate that the FFQ is a useful tool for ranking children according to food items/groups intake. The low respondent burden and relative simplicity of the FFQ makes it suitable for use in large cohort studies of 9–10 year-old children in New Zealand.

## 1. Introduction

Dietary intake is an important determinant of nutritional status and health during both childhood and adulthood [1,2]. Measuring intake of foods and/or food groups (e.g., fruit, meat, beverages, cake, *etc*.) is essential as there are moderate to strong links between dietary patterns and increased risk of chronic diseases such as cardiovascular disease, hypertension and type 2 diabetes [3]. It has been identified that dietary patterns formed in early stages of life can track into adulthood. Therefore, it is important to accurately measure children’s dietary intake to identify diet-related behaviours and potential areas for change in order to optimise current and future health [4,5]. Food frequency questionnaires (FFQs) have been the most frequently used dietary assessment tool in large-scale epidemiological studies and also nutrition-related studies [6,7]. FFQs are practical, easy to administer and inexpensive tools that do not affect food intake patterns [8,9]. Also, FFQs can assess habitual dietary patterns with a single administration [10]. Although various studies have used FFQs of different lengths to assess the whole diet [11,12,13,14,15], when measuring a single nutrient or food group is of interest, short FFQs are useful. Also, short FFQs are appropriate tools to explore factors associated with changes in dietary patterns of the population [16].

FFQs are useful tools in children 9 years old and above to accurately assess their dietary intake [17]. It has been also shown that school-age children are the most accurate reporter of their dietary intake using an FFQ, as they are more independent than their younger counterparts and their parents may not know what their children eat and drink when away from home [18]. However, cognitive characteristics of children need to be taken into consideration when assessing their dietary intake. Children might have limitations in describing and quantifying portion sizes, knowledge about food and its preparation [19]. Therefore, using a simple and short dietary assessment tool that can accurately rank children’s food intake is necessary [20]. Also, it has been reported that frequency of consumption seems to have a greater impact on dietary intake than portion sizes [9]. Therefore, short non-quantitative FFQs are considered useful and appropriate in studies of children. 

Many studies have shown that reliable and valid non-quantitative short FFQs can be a pragmatic way to assess the usual intakes of a particular food or food groups in children [21,22,23,24,25,26,27,28]. Using a valid and reliable FFQ is critical [9], since an FFQ with low validity may attenuate associations between dietary intakes and disease. Therefore, all newly developed or modified FFQs need to be tested for reproducibility and validity [9,29,30]. As there is no short, valid, and up-to-date FFQ (*i.e.*, validated within the past five years) available for use in 9–10 year-old children in New Zealand, the aim of this study was to develop a short non-quantitative FFQ, then determine its reproducibility and relative validity in this age group. 

## 2. Materials and Methods

In this section, development of the Physical activity, Exercise, Diet And Lifestyle Study FFQ (PEDALS FFQ), its reproducibility and validity procedures are discussed. 

### 2.1. FFQ Development

The PEDALS FFQ is a modified version of a previously validated 72-item New Zealand Adolescent FFQ (NZAFFQ) [24]. Considering the age of our population of interest (*i.e.*, 9–10 year-old children) some changes were applied to the NZAFFQ, as it was validated in an older population of 14–18 year-old adolescents. Therefore, some of the wordings were rephrased (e.g., ‘Standard milk’ changed to ‘Standard milk (blue)’ and ‘Trim milk’ changed to ‘Trim milk (green)’ to make them better understood by 9–10 year-old children. Standard milk is full fat milk (blue top) and trim milk is low fat/skimmed milk (green top). Tea, ice tea, coffee and alcoholic beverages were removed and new food items (‘Tomato sauce and ketchup’ and ‘Jam and honey’) were added, to cover commonly consumed food items by 9–10 year-old New Zealand children. Thirteen fruits from the NZAFFQ questionnaire were grouped together as one item called ‘Fruits’ to be added to the PEDALS FFQ. Similarly, 22 vegetables from the NZAFFQ were aggregated into one group called ‘Vegetables’ in the PEDALS FFQ. Further, two nutritionists and a dietitian reviewed the key results of the 2002 National Children’s Nutrition Survey to ensure the PEDALS FFQ covered major contributors to the diet of children [31]. The final PEDALS FFQ was made up of 28 food items/groups. The foods were included either as single food items (e.g., ‘Cheese’), or lists of foods, which were nutritionally similar (e.g., ‘Processed meat’). A complete list of foods included in the PEDALS FFQ (28-item) is found in the right column of Table 1. These 28 food items/groups were then aggregated into 23 (left column in Table 1) to assess the reproducibility and validity of the PEDALS FFQ. For example ‘Vegetables’ and ‘Potato’ were grouped together as one item. ‘Fruit juice’, ‘Diet fizzy drinks’, and ‘Fizzy drinks’ were aggregated into one group called ‘Non-dairy drinks’. ‘Potato chips’ and ‘Hot chips’ questions were combined to form ‘Salty snacks’ group. Also, ‘Snack bars’, ‘Chocolate and chocolate bars’ grouped together as ‘Sweet snacks’ (Table 1). To determine the frequency of intake children were asked ‘How many times a week do you usually eat or drink?’ and children could select one of the seven frequency options including; ‘Never’, ‘Less than once a week’, ‘Once a week’, ‘2–4 days a week’, ‘5–6 days a week’, ‘Every day, once a day’ and ‘Every day, more than once’. Finally, three experts (two nutritionists and a dietitian) reviewed the FFQ before formal testing to ensure all frequently consumed foods by children were included and to help improve its face validity. 

### 2.2. Data Collection

This study has examined whether the PEDALS FFQ is a reproducible and valid tool to assess dietary intake of 9–10 year-old children in New Zealand. The study was approved by the University of Otago Ethics Committee (Ref No. 14/227). Firstly, letters outlining the study were sent to the principals of a convenience sample of four public primary schools in Dunedin, of which three agreed to take part. One of the schools did not take part in the study as they could not accommodate participation during the study time frame. All the participating schools were high decile schools. A study team visited each of the schools on three occasions and a convenience sample of children was selected between August and October 2014. The first school visit was a study information visit, where researchers explained the purpose of the study and procedures involved to all 9 to 10 year-old children. During this visit study packs containing information and consent forms were given to interested children to take home to their parents/primary caregiver. To be involved in the study the consent of parents and the assent of children were both necessary, and children were asked to return the signed forms to school. Approximately one to two weeks later, a second school visit was scheduled at the end of the school day for those who had returned both the consent and assent forms. Parents were also invited to attend if they wished. During this visit, study researchers provided children instructions on how to complete the PEDALS FFQ, and children then completed the FFQ for the first time. Children’s height and weight were measured in this session. At this visit a detailed explanation was provided to children on how to complete the four-day estimated food diary (4DEFD), and researchers went over a typical example day of diet recording with children. Children were then asked to complete the food diary on four non-consecutive assigned days, pre-determined by the researchers, over the following two weeks (three weekdays and one weekend day). During this period researchers phoned and/or texted parents four times (before each assigned day for food diary completion) to remind them of the food diary completion days and answer any questions parents or children had about completing the food diaries. At the third school visit, children completed the PEDALS FFQ for the second time, approximately two weeks (range 12 to 14 days) after the first administration. Food diaries were checked for understanding and completeness. The choice of the two-week interval between two FFQ administrations was due to the fact that longer time intervals may result in true changes in dietary intake and also variation in response [32].

### 2.3. Validation Reference: 4DEFD 

Doubly labeled water (DLW) is the gold standard method of measuring total energy expenditure in human. However, due to high costs, burden on participants, requirements of high technical skills, and special facilities needed, DLW is rarely used [33]. Compared to the DLW method, 24-h multiple pass recall and weighed food diaries are considered acceptable accurate methods to measure children’s dietary intake. We chose to use an estimated 4DEFD as our reference method, since first of all, the collection of three to seven days of food diaries is normally adequate to assess food group intake [9]. Secondly, weighed food diaries would place a large burden on our 9–10 year-old children. The 4DEFD was completed between the first and second FFQ administrations. Results of previous research show that children aged 9 years and above are able to accurately self-report their dietary intake [34,35,36]. Also, to further facilitate accurate recording, an example of a typical day of diet recording was provided in the 4DEFD. The food diary was structured into seven different time slots, as follows: 6 a.m. to 9 a.m., 9 a.m. to 12 noon, 12 noon to 2 p.m., 2 p.m. to 5 p.m., 5 p.m. to 8 p.m., 8 p.m. to 10 p.m., and 10 p.m. to 6 a.m. For each assigned day children were asked to answer the following questions regarding their food and beverage consumption; mealtime, venue, with whom the participant ate, amount of both foods and beverages they consumed, where did they get the food and who did they get the food from. In addition, a ‘Diet Assessment Photos’ booklet which included photos of various portion sizes of commonly consumed food and beverages in New Zealand (e.g., pasta, broccoli, chicken) was provided to each child to assist them in recording food quantities. Also, the booklet included photos of a ruler and a diameter circle to help children to estimate the portion size of food items such as cheese and biscuits [37]. Children were asked to obtain help from their parents/primary caregiver, if needed, when completing their food diaries. Space was also provided in the 4DEFD for parents to record homemade recipes, if any were used, for those assigned days. All 4DEFD were checked for completeness during the third school visit and children were asked for further information, where necessary.

### 2.4. Anthropometric Measurements

Height was measured without shoes to the nearest 0.1 cm using a portable stadiometer (Holtain Limited, Crymych, United Kingdom) with children’s head positioned in the Frankfort plane. Measurements were taken in duplicate or triplicate if the first two measurements differed by more than 0.5 cm. Body weight was also measured in duplicate, in light clothing and no footwear to the nearest 0.1 kg with a portable digital scale (Seca alpha, Model 770, Hamburg, Germany). Weight status was defined using the International Obesity Taskforce age- and sex-specific BMI cut-offs for children and adolescents [38,39]. BMI *z*-scores were also determined, using the Centers for Disease Control and Prevention (CDC) method [40]. 

### 2.5. Data Entry

All data for both administrations of the PEDALS FFQ and the 4DEFD were entered into a Microsoft Excel Spreadsheet and were checked by a trained nutritionist. Food items from the 4DEFD were matched to items from the PEDALS FFQ. As an example, porridge from 4DEFD was assigned to the ’Breakfast cereals’ group in the FFQ. Mixed foods were each allocated a proportion of the meal and then assigned to their relevant food or food groups from the FFQ. For example, Teriyaki chicken sushi was considered as the two following food groups in the PEDALS FFQ: ‘Other meats’ and ‘Rice’. Also, if a child had a ham sandwich with carrot and cheese slices for lunch, this was considered as ‘Bread’, ‘Vegetables’, ‘Processed meat’ and ‘Cheese’ for comparison to the FFQ. For assigning the frequency to each of the food items from the 4DEFD, a frequency of ‘1’ was given to each food item, if it was consumed even more than once during a specific food occasion e.g., if a meal contained both carrots and broccoli. Frequency of intake of food and beverages from the FFQ were based on weekly frequencies, whereas, data from the 4DEFD were based on frequency over four days. Therefore, to provide comparable data, the 4DEFD data were converted into a weekly intake. As each 4DEFD included three weekdays and a weekend day, to provide an estimate of weighted average weekly frequencies, average frequency of food intake was divided by three and multiplied by five for weekdays and multiplied by two for weekend days. 

### 2.6. Statistical Analysis

The reproducibility of the PEDALS FFQ was assessed by administrating the FFQ twice, within a specified time interval (two weeks), to the same group of participants. Previous studies have used different statistical tests such as correlation coefficients to assess the reproducibility [24,41,42]. Correlation coefficients show the degree to which two administrations of the FFQ are associated, but not the agreement [43]. Therefore, it is advisable to use the correlation coefficient in conjunction with other methods. To determine the reproducibility of the PEDALS FFQ, both the Spearman’s correlation coefficient (SCC) and intraclass correlation coefficients (ICCs) were calculated. The ICC is considered as the most useful test to determine the agreement between repeated FFQs, taking into account an individual’s ranking based on the food group intake. The Cohen’s cut-offs were used to interpret the SCC values. According to these cut-offs, *r* = ±0.5 is considered strong, *r* = ±0.30 moderate and *r* = ±0.10 as weak [44]. The following definitions are used to interpret the ICC values; ICC ≤ 0.40 is ‘poor’, 0.41 < ICC < 0.59 is ‘fair’, 0.60 < ICC < 0.74 is ‘good’ and 0.75 < ICC < 1.00 is ‘excellent’ [45]. Cross-classification analysis, which is more informative than a correlation coefficient, was used to rank children into thirds with regard to their intake. Therefore, the proportion of participants whom were correctly classified (CC%) into the same thirds or grossly misclassified (GC%) into the extreme thirds was also determined. To determine the relative validity of the PEDALS FFQ against the 4DEFD, SCCs were calculated. Correlation coefficients above 0.30 are considered as acceptable in FFQ validation studies [7,44]. Stata 11.2 (StataCorp, College Station, TX, USA) was used to run the statistical analysis. For data analysis, the 28 food items/groups from the FFQ were aggregated into 23 food items/groups, taking into account the similar nutrition content and/or the way these foods are consumed. Table 1 shows the aggregation of the food items/groups of the PEDALS FFQ.

## 3. Results

Overall, 176 children and their parents were invited to participate. Fifty-six (32.0%) agreed to be involved in this study (23, 17, and 16 children from the first, second and third school, respectively). Fifty children (89.3%) completed both FFQs and the 4DEFD. The mean age of participants was 9.40 ± 0.49 years. No significant differences were observed between boys and girls in demographic characteristics. In total 22.0% of participants were overweight and 2.0% were obese. Participants’ mean BMI *z*-score was 0.45 ± 0.86.

### 3.1. Reproducibility: PEDALS FFQ One vs. PEDALS FFQ Two

The median SCC was 0.66 and ranged from 0.40 for ‘Processed meat’ to 0.82 for ‘Lollies’ and ‘Non-dairy drinks’, with all food groups achieving correlations above 0.40 (Table 2). The ICC ranged from 0.35 for ‘Sweet snacks’ to 0.78 for ‘White bread’ and more than half of the food items/groups (52.2%) had an ICC more than 0.5. The majority of the food items/groups (73.9%) had levels of gross misclassification less than 10 percent. There was no misclassification for ‘Non-dairy drinks’, ‘Salty snacks’ and ‘Lollies’ (Table 2).

Comparison of results obtained from the PEDALS FFQ with the previously validated NZAFFQ in adolescents in New Zealand [24], showed that most of the food items have similar SCCs and ICCs. Some exceptions were as follows; in the PEDALS FFQ ‘Fruits’ and ‘Vegetables’ had a higher SCC, whereas, ‘Standard milk’ and ‘Brown bread’ had lower SCC compared to the NZAFFQ (Table 2).

### 3.2. Relative Validity: PEDALS FFQ vs. 4DEFD

Table 3 shows that the majority (69.6%) of the food items/groups (16 out of 23) had a SCC equal to or greater than 0.3, ranging from −0.11 for the ‘Tomato sauce and ketchup’ food group to 0.58 for the ‘Peanut butter and Nutella’ food group, which is considered a moderate to strong correlation [44,46].

The proportion of children who were correctly classified (CC%) into the same thirds varied from 34.0% for ‘Pasta’ to 54.0% for ‘Breakfast cereals’. For some of the food items/groups the distribution of frequencies of consumption did not allow for categorisation into thirds. Therefore, these food items/groups were presented based on the Yes/No categories. The proportion of children who were correctly classified into Yes/No categories ranged from 24.0% for ‘Tomato sauce and ketchup’ to 70.0% for ‘Peanut butter and Nutella’. 

## 4. Discussion

The short-term reproducibility and relative validity of the PEDALS FFQ are discussed in the following section. 

### 4.1. Reproducibility

Our results showed that the PEDALS FFQ performed reasonably well in terms of reproducibility. Considering the definition of ICC values by Cicchetti [45], the results of the study showed that 19 out of 23 food items/groups in the PEDALS FFQ had ‘fair’ to ‘excellent’ ICC values. Further, the SCC which measure reproducibility of the FFQ food items/groups in the PEDALS FFQ fell within or above the common range of SCC of 0.5–0.7 for most food items/group [7]. The median SCC was 0.66, which is considered as good for an FFQ [9]. Twelve out of 23 food items/groups had strong correlation coefficients (0.5–0.7) and eight food groups had SCC of more than 0.7. Only ‘Processed meat’, ‘Other meats’ and ‘Pasta’ had a SCC less than 0.5.

Our SCC values regarding ‘Fruits’ (0.66 *vs.* 0.62) and ‘Vegetables’ (0.66 *vs.* 0.70) were similar to those from a study conducted in Norwegian children (mean age 12.9 years) [47]. It has been shown that often eaten food items such as fruits and vegetables have higher correlations compared to occasionally eaten food items such as pizza [27]. Compared to our findings, Vereecken *et al.* reported similar SCC for the following food groups in 11–12 year-old Belgian children; ‘Fruits’ (0.66 *vs.* 0.69), ‘Trim milk’ (0.70 *vs.* 0.68), ‘Standard milk’ (0.57 *vs.* 0.59), and ‘Breakfast cereals’ (0.75 *vs.* 0.70) [28]. On the other hand, Vereecken *et al.* found lower SCC for ‘Trim milk’ (0.65 *vs.* 0.70) and ‘White bread’ (0.40 *vs.* 0.77) in Italian children. However, ‘Breakfast cereals’ had almost the same SCC compared to our findings (0.73 *vs.* 0.75) [28]. In a study among 11–12 year-old Flemish children, crisps had the same SCC compared to ‘Salty snacks’ in our study [48]. Consistently, Vereecken *et al.* [28] found a similar SCC for crisps (0.63) in both Belgian and Italian children compared to our results (‘Salty snacks’; 0.74). It has been reported that snack foods such as potato crisps, corn chips and vegetable/grain crisp are the most significant contributors to the energy intake of children in New Zealand on schooldays and children can easily access hot chips during the week [49]. Therefore, snack foods as a commonly consumed food item in children are easier to be recalled compared to foods eaten more sporadically. The range of SCC values in our study (0.40–0.82) were similar to those which were reported in studies conducted in 11–12 year-old Belgian (0.49–0.79) and Italian children (0.40–0.83) [28]. Consistent with our results, findings from an FFQ reproducibility study of 48 children aged 5–9 years old in New Zealand [50] showed almost the same SCC values for the following food items/groups; ‘Vegetables’ (0.66 *vs.* 0.67), ‘Fruits’ (0.66 *vs.* 0.71), ‘Fish’ (0.67 *vs.* 0.75) and ‘Rice’ (0.70 *vs.* 0.85). Also, Metcalf *et al.* reported similar SCC values for ‘Vegetables’ (0.66 *vs.* 0.76) and ‘Fish’ (0.67 *vs.* 0.62) food items/groups in 10–14 year-old children [50]. As food group management was different in the Metcalf *et al.* study, it was difficult to reasonably compare other food items/groups. 

### 4.2. Relative Validity 

Several studies have validated FFQs among children and many of these studies validated their FFQs in terms of nutrients [51,52,53,54,55]. However, the focus of the current study was on the frequency of consumption of key food items/groups. 

In our study, the majority of the food items/groups (69.5%) had a SCC between 0.3 and 0.5, while seven food items yielded correlations below 0.30. In particular, the PEDALS FFQ was less accurate in estimating the intake of ‘Other meats’, ‘Fish’, ‘Pasta’, ‘Biscuits’, ‘Lollies’ and ‘Tomato sauce’ food items/groups. There are several possible explanations for this level of validity for these food items/groups. Children might eat some food items less frequently and not on a regular basis, which can lead to a lower relative validity for that food item. In line with this, the SCC values found in this study tended to be lower for less frequently consumed food items such as fish. The mixed dishes in the 4DEFD were divided into their components and assigned to their matching food items from the FFQ. As a result, foods often consumed as part of the mixed dishes such as mince, chicken and fish may either be forgotten by children or miscoded in the food diaries due to insufficient information [49,56]. Also, children might not be able to recall the frequency of some food items such as ‘Tomato sauce and ketchup’, which are usually consumed as a side of main dishes (e.g., with hot chips or pizza). Further, some of the food items can be considered as less healthy and children could underreport these food items due to their social undesirability [57]. Although the SCC value was very low for ‘Pasta’ during the first administration, it did reasonably well during the second administration (0.07 *vs.* 0.34). Also, it should be taken into account that correlation coefficients from FFQs generally have lower values among children than adults [58].

Based on our results, the ‘Peanut butter and Nutella’ food group had the highest SCC (*r* = 0.58), followed by ‘Standard milk’ (*r* = 0.50), ‘Breakfast cereals’ (*r* = 0.47), ‘Yoghurt’ (0.46) and ‘Trim milk’ (0.45). Vereecken and Maes [48] in a sample of 10–17 year-old children and adolescents found that semi-skimmed milk (0.65) and whole-fat milk (0.64) had the highest SCC. A study in 9 year-old Norwegian children also showed that low-fat milk (0.67) and full-fat milk (0.66) had the highest SCC [27]. Breakfast cereals are the most commonly consumed breakfast food (57%) among children in New Zealand, followed by bread and toast (35%), beverages such as Milo (14%), fruit juices (11%) and milk (8%) [59]. Children usually consume milk with breakfast cereals or as chocolate-flavoured drinks such as Milo/hot chocolate. Therefore, milk as part of a more regular eating practice is easily remembered by children compared to other food items in the FFQ.

In our analysis CC% (based on tertiles) between frequency of intakes from the PEDALS FFQ one and the 4DEFD ranged from 34.0% for ‘Pasta’ to 70.0% for the ‘Peanut butter and Nutella’ food group. In a study conducted by Bel-Serrat [25] CC% ranged from 27.7% for meat to 52.5% for sweetened milk among 6–9 year-old European children. These variations could be due to the differences in food group management and also food intake differences across countries. 

Given the differences in designing FFQs, time between first and second administration, statistical methods, data management and age range of the study subjects, comparing the results with other studies regarding the reproducibility and validity should be done with caution. These differences make it difficult for an exact comparison of the studies. Also, correlation coefficients are affected by the mode of administration, age, sex and ethnicity of the population [60]. 

### 4.3. Strength and Limitations

The PEDALS FFQ was adopted from a previously validated FFQ in adolescents in New Zealand (NZAFFQ), which can be a useful tool in future interventional or epidemiological studies to understand children’s specific food group intake and its link with weight status or other markers of health. There were, however, a few limitations with this study. Firstly, our participants were 9–10 year-old children. It is mentioned that children ≥9 years are the most accurate reporters of their own dietary intake, since parent(s)/primary caregiver may not be able to accurately report what their children eat or drink while they are away from home [18]. However, factors such as forgetfulness and limited knowledge of food items in children can cause food underestimation [10,61,62]. To minimize this limitation, we asked parent(s)/primary caregiver to provide help with completion of the food diaries. Secondly, although no sharp cut-off for an optimal sample size is defined when considering the validity of an FFQ, a sample size of at least 50 is desirable [7,9]. However, Willett has suggested a sample size of 100 to 200 as reasonable for validation studies [32]. 

## 5. Conclusions

FFQs for use in epidemiological studies must be designed for a specific population group and include culturally relevant food items [63]. The PEDALS FFQ was designed as a self-administered short and non-quantitative FFQ to assess usual dietary intake of 9–10 year-old children in New Zealand, which took approximately 15 min to be completed. The results indicate that the PEDALS FFQ gives reproducible estimates of food group intakes. With regard to validity analysis, food items such as ‘Peanut butter and Nutella’, ‘Standard milk’, ‘Breakfast cereals’, ‘Yoghurt’, ‘Trim milk’, ‘White bread’, ‘Brown/wholemeal bread’ and ‘Sweet snacks’ had the highest SCC values. Therefore, the PEDALS FFQ could be used as a valid tool to rank children according to the frequency consumption of these food items/groups. In addition, the low respondent burden and logistical implications of the short FFQ makes it a useful tool for future studies among 9–10 year-old children in New Zealand.

## Figures and Tables

**Table 1:** Food grouping of the PEDALS FFQ for the analysis.

	Food Items/Groups List in the PEDALS FFQ after Aggregation (23 Items)	N ^$^	Complete List of Food Items/Groups List in the PEDALS FFQ before Aggregation (28 Items)
1	Fruits	1	Fruits
2	Vegetables	2	Vegetables (excluding potato)
		2	Potato (such as mashed, boiled)
3	Trim milk (green) (including on cereals, milo, hot chocolate) ^#^	3	Trim milk (green) (including on cereals, milo, hot chocolate)
4	Standard milk (blue) (including on cereals, milo, hot chocolate) *	4	Standard milk (blue) (including on cereals, milo, hot chocolate)
5	Cheese	5	Cheese
6	Yoghurt	6	Yoghurt
7	Ice-cream	7	Ice-cream
8	Processed meat (such as meat pies, sausage, sausage roll, salami, luncheon, bacon, ham)	8	Processed meat (such as meat pies, sausage, sausage roll, salami, luncheon, bacon, ham)
9	Other meats (such as mince, beef, chicken)	9	Other meats (such as mince, beef, chicken)
10	Fish (including canned tuna or salmon, fish cakes, fish fingers, fish pie, battered fish)	10	Fish (including canned tuna or salmon, fish cakes, fish fingers, fish pie, battered fish)
11	Non-dairy drinks	11	Fruit juice (such as orange juice, apple juice, Raro, Refresh, Keri, Twist, Ribena)
		11	Diet fizzy drinks (such as Diet Coke, Pepsi Max, Sprite Zero and any other light or sugar free varieties)
		11	Fizzy drinks (such as Coke, Pepsi, Sprite, L&P, Fanta, Ginger Beer)
12	Breakfast cereals	12	Breakfast cereals
13	White bread	13	White bread
14	Brown/wholemeal bread	14	Brown/wholemeal bread
15	Rice, rice based dishes	15	Rice, rice based dishes
16	Pasta (such as spaghetti, macaroni), noodles	16	Pasta (such as spaghetti, macaroni), noodles
17	Salty snacks	17	Potato chips, potato snacks, corn chips
		17	Hot chips, wedges, French fries
18	Biscuits, cakes, muffins, doughnuts, fruit pies	18	Biscuits, cakes, muffins, doughnuts, fruit pies
19	Lollies ^$$^	19	Lollies
20	Sweet snacks	20	Snack bars (such as muesli bar, fruit bar, rice bubble bar)
		20	Chocolate, chocolate bars
21	Tomato sauce, ketchup	21	Tomato sauce, ketchup
22	Peanut butter, Nutella	22	Peanut butter, Nutella
23	Jam, honey	23	Jam, honey

PEDALS FFQ: Physical activity, Exercise, Diet And Lifestyle Study food frequency questionnaire; ^$^ Food items/groups with the same number (N) in right column, are aggregated to form a new group in left column (e.g., ‘Vegetables (excluding potato)’ and ‘Potato (such as mashed, boiled)’ were grouped together as one, called ‘Vegetables’; * ‘Standard milk (blue)’: Full fat milk (blue top); ^#^ ‘Trim milk (green)’: Low fat/skimmed milk (green top); ^$$^ ‘Lollies’: confectioneries/candies e.g., Jelly beans, wine gums, marshmallows, liquorice, Minties.

**Table 2:** Reproducibility of the PEDALS FFQ (*n* = 50).

Food Group	SCC	ICC	CC%	GM%	SCC (NZAFFQ)	ICC (NZAFFQ)
Fruits	0.66	0.63	58	8	0.57	0.83
Vegetables ^†^	0.66	0.60	60	2	0.42	-
Trim milk (green)	0.70	0.54	70	6	0.87	0.79
Standard milk (blue)	0.57	0.43	70	6	0.83	0.78
Cheese	0.57	0.40	60	10	0.67	0.58
Yogurt	0.74	0.48	64	2	0.79	0.84
Ice cream	0.61	0.64	68	12	0.64	0.79
Processed meat	0.40	0.38	52	14	-	-
Other meats	0.48	0.43	44	12	-	-
Fish	0.67	0.43	62	8	0.76	0.67
Non-dairy drinks ^††^	0.82	0.75	80	0	-	-
Breakfast cereals	0.75	0.72	82	2	0.78	0.74
White bread	0.77	0.78	72	4	0.78	0.64
Brown/wholemeal bread	0.58	0.48	56	8	0.80	0.72
Rice, rice based dishes	0.70	0.73	68	6	-	-
Pasta (such as spaghetti, macaroni), noodles	0.45	0.44	48	10	-	-
Salty snacks ^‡^	0.74	0.73	66	0	-	-
Biscuits, cakes, muffins, doughnuts, fruit pies	0.59	0.38	56	8	0.65	0.62
Lollies	0.82	0.75	72	0	-	-
Sweet snacks ^§^	0.63	0.35	70	10	-	-
Tomato sauce, ketchup	0.65	0.41	60	4	-	-
Peanut butter, Nutella	0.75	0.70	66	2	0.79	0.73
Jam, honey	0.77	0.72	64	2	-	-

PEDALS FFQ: Physical activity, Exercise, Diet And Lifestyle Study food frequency questionnaire; SCC: Spearman’s correlation coefficient; ICC: intraclass correlation coefficient; CC%: percentage correct classified; GM%: percentage grossly misclassified; NZAFFQ: New Zealand Adolescent Food Frequency Questionnaire; ^†^ ‘Vegetables’ and ‘Potato’ questions combined; ^††^ ‘Fruit juice’ and ‘Diet fizzy drinks’ and ‘Fizzy drinks’ questions combined; ^‡^ ‘Potato chips’ and ‘Hot chips’ questions combined; ^§^ ‘Snack bars’ and ‘Chocolate and chocolate bars’ questions combined.

**Table 3:** Relative validity of the PEDALS FFQ (*n* = 50).

Food Group	SCC	CC% (PEDALS FFQ One)	GM%	SCC (NZAFFQ)
Fruits	0.28	38	16	0.34
Vegetables ^†^	0.31	44	16	0.37
Trim milk (green)	0.45	60 ^¶^	40 ^¶^	0.59
Standard milk (blue)	0.50	44	6	0.70
Cheese	0.34	42	10	0.40
Yogurt	0.46	48	10	0.46
Ice cream	0.30	44	14	0.42
Processed meat	0.36	42	14	-
Other meats	0.13	48	18	-
Fish	0.14	52 ^¶^	48 ^¶^	0.34
Non-dairy drinks ^††^	0.39	48	14	-
Breakfast cereals	0.47	54	10	0.67
White bread	0.42	48	10	0.40
Brown/wholemeal bread	0.41	46	6	0.36
Rice, rice based dishes	0.38	44	10	-
Pasta (such as spaghetti, macaroni), noodle	0.07	34	26	-
Salty snacks ^‡^	0.31	42	14	-
Biscuits, cakes, muffins, doughnuts, fruit pies	0.26	44	16	0.56
Lollies	0.25	38	14	-
Sweet snacks ^§^	0.40	50	14	-
Tomato sauce, ketchup	−0.11	24 ^¶^	76 ^¶^	-
Peanut butter, Nutella	0.58	70 ^¶^	30 ^¶^	0.37
Jam, honey	0.37	52	14	-

PEDALS FFQ: Physical activity, Exercise, Diet And Lifestyle Study food frequency questionnaire; SCC: Spearman correlation coefficient; CC%: percentage correct classified; GM%: percentage grossly misclassified; NZAFFQ: New Zealand Adolescent Food Frequency Questionnaire; ^†^ ‘Vegetables’ and ‘Potato’ questions combined; ^††^ ‘Fruit juice’ and ‘Diet fizzy drinks’ and ‘Fizzy drinks’ questions combined; ^‡^ ‘Potato chips’ and ‘Hot chips’ questions combined; ^§^ ‘Snack bars’ and ‘Chocolate and chocolate bars’ questions combined; ^¶^ Based on Yes/No category.

## Abbreviations

The following abbreviations are used in this manuscript:

FFQ	Food frequency questionnaire
PEDALS	Physical activity, Exercise, Diet And Lifestyle Study FFQ
4DEFD	Four-day estimated food diary
SCC	Spearman’s correlation coefficients
ICC	Intraclass correlation coefficients
NZAFFQ	New Zealand Adolescent FFQ
CC%	Percentage correctly classified
GC%	Percentage grossly misclassified

## References

[1] Mikkilä V., Räsänen L., Laaksonen M.M.L., Juonala M., Viikari J., Pietinen P., Raitakari O.T. (2009). Long-term dietary patterns and carotid artery intima media thickness: The Cardiovascular Risk in Young Finns Study. Br. J. Nutr..

[2] Victora C.G., Adair L., Fall C., Hallal P.C., Martorell R., Richter L., Sachdev H.S. (2008). Maternal and child undernutrition: Consequences for adult health and human capital. Lancet.

[3] Dietary Guidelines Advisory Committee SCIENTIFIC Report of the 2015 Dietary Guidelines Advisory Committee. http://health.gov/dietaryguidelines/2015-scientific-report/.

[4] Mikkilä V., Räsänen L., Raitakari O.T., Pietinen P., Viikari J. (2005). Consistent dietary patterns identified from childhood to adulthood: The cardiovascular risk in Young Finns Study. Br. J. Nutr..

[5] Northstone K., Emmett P.M. (2008). Are dietary patterns stable throughout early and mid-childhood? A birth cohort study. Br. J. Nutr..

[6] Hammond J., Nelson M., Chinn S., Rona R.J. (1993). Validation of a food frequency questionnaire for assessing dietary intake in a study of coronary heart disease risk factors in children. Eur. J. Clin. Nutr..

[7] Cade J., Thompson R., Burley V., Warm D. (2002). Development, validation and utilisation of food-frequency questionnaires—A review. Public Health Nutr..

[8] Andersen L.F., Johansson L., Solvoll K. (2002). Usefulness of a short food frequency questionnaire for screening of low intake of fruit and vegetable and for intake of fat. Eur. J. Public Health.

[9] Willett W., Larent E., Willett W. (1998). Reproducibility and validity of food-frequency questionnaires. Nutritional Epidemiology.

[10] Kobayashi T., Kamimura M., Imai S., Toji C., Okamoto N., Fukui M., Date C. (2011). Reproducibility and validity of the food frequency questionnaire for estimating habitual dietary intake in children and adolescents. Nutr. J..

[11] Andersen L.F., Nes M., Lillegaard I.T., Sandstad B., Bjørneboe G.E., Drevon C.A. (1995). Evaluation of a quantitative food frequency questionnaire used in a group of Norwegian adolescents. Eur. J. Clin. Nutr..

[12] Rockett H.R., Breitenbach M., Frazier A.L., Witschi J., Wolf A.M., Field A.E., Colditz G.A. (1997). Validation of a youth/adolescent food frequency questionnaire. Prev. Med..

[13] Subar A.F., Thompson F.E., Kipnis V., Midthune D., Hurwitz P., McNutt S., McIntosh A., Rosenfeld S. (2001). Comparative validation of the Block, Willett, and National Cancer Institute food frequency questionnaires: The Eating at America’s Table Study. Am. J. Epidemiol..

[14] Schatzkin A., Kipnis V., Carroll R.J., Midthune D., Subar A.F., Bingham S., Schoeller D.A., Troiano R.P., Freedman L.S. (2003). A comparison of a food frequency questionnaire with a 24-h recall for use in an epidemiological cohort study: Results from the biomarker-based Observing Protein and Energy Nutrition (OPEN) study. Int. J. Epidemiol..

[15] Ambrosini G.L., O’Sullivan T.A., de Klerk N.H., Mori T.A., Beilin L.J., Oddy W.H. (2011). Relative validity of adolescent dietary patterns: A comparison of a FFQ and 3 d food record. Br. J. Nutr..

[16] Osler M., Heitmann B.L. (1996). The validity of a short food frequency questionnaire and its ability to measure changes in food intake: A longitudinal study. Int. J. Epidemiol..

[17] Collins C.E., Watson J., Burrows T. (2010). Measuring dietary intake in children and adolescents in the context of overweight and obesity. Int. J. Obes..

[18] Burrows T.L., Truby H., Morgan P.J., Callister R., Davies P.S.W., Collins C.E. (2013). A comparison and validation of child versus parent reporting of children’s energy intake using food frequency questionnaires versus food records: Who’S an accurate reporter?. Clin. Nutr. Edinb. Scotl..

[19] Livingstone M.B.E., Robson P.J. (2000). Measurement of dietary intake in children. Proc. Nutr. Soc..

[20] Flood V.M., Wen L.M., Hardy L.L., Rissel C., Simpson J.M., Baur L.A. (2014). Reliability and validity of a short FFQ for assessing the dietary habits of 2–5-year-old children, Sydney, Australia. Public Health Nutr..

[21] Lazarou C., Panagiotakos D.B., Kouta C., Matalas A.L. (2009). Dietary and other lifestyle characteristics of Cypriot school children: Results from the nationwide CYKIDS study. BMC Public Health.

[22] Magarey A., Golley R., Spurrier N., Goodwin E., Ong F. (2009). Reliability and validity of the Children’s Dietary Questionnaire; a new tool to measure children’s dietary patterns. Int. J. Pediatr. Obes..

[23] Kiwanuka S.N., Astrøm A.N., Trovik T.A. (2006). Sugar snack consumption in Ugandan schoolchildren: Validity and reliability of a food frequency questionnaire. Community Dent. Oral Epidemiol..

[24] Wong J.E., Parnell W.R., Black K.E., Skidmore P.M.L. (2012). Reliability and relative validity of a food frequency questionnaire to assess food group intakes in New Zealand adolescents. Nutr. J..

[25] Bel-Serrat S., Mouratidou T., Pala V., Huybrechts I., Börnhorst C., Fernández-Alvira J.M., Hadjigeorgiou C., Eiben G., Hebestreit A., Lissner L. (2014). Relative validity of the Children’s Eating Habits Questionnaire-food frequency section among young European children: The IDEFICS Study. Public Health Nutr..

[26] De Keyzer W., Dekkers A., Van Vlaslaer V., Ottevaere C., Van Oyen H., De Henauw S., Huybrechts I. (2013). Relative validity of a short qualitative food frequency questionnaire for use in food consumption surveys. Eur. J. Public Health.

[27] Lillegaard I.T., Overby N.C., Andersen L.F. (2012). Evaluation of a short food frequency questionnaire used among Norwegian children. Food Nutr. Res..

[28] Vereecken C.A., Rossi S., Giacchi M.V., Maes L. (2008). Comparison of a short food-frequency questionnaire and derived indices with a seven-day diet record in Belgian and Italian children. Int. J. Public Health.

[29] Raitakari O.T., Juonala M., Kähönen M., Taittonen L., Laitinen T., Mäki-Torkko N., Järvisalo M.J., Uhari M., Jokinen E., Rönnemaa T. (2003). Cardiovascular risk factors in childhood and carotid artery intima-media thickness in adulthood: The Cardiovascular Risk in Young Finns Study. JAMA.

[30] Saland J.M. (2007). Update on the metabolic syndrome in children. Curr. Opin. Pediatr..

[31] Ministry of Health (2003). NZ Food NZ Children: Key Results of the 2002 National Children’s Nutrition Survey.

[32] Willett W., Willett W., Lenart E. (2013). Reproducibility and validity of food frequency questionnaires. Nutritional Epidemiology.

[33] Burrows T.L., Martin R.J., Collins C.E. (2010). A systematic review of the validity of dietary assessment methods in children when compared with the method of doubly labeled water. J. Am. Diet. Assoc..

[34] Baranowski T., Dworkin R., Henske J.C., Clearman D.R., Dunn J.K., Nader P.R., Hooks P.C. (1986). The accuracy of children’s self-reports of diet: Family Health Project. J. Am. Diet. Assoc..

[35] Baranowski T., Domel S.B. (1994). A cognitive model of children’s reporting of food intake. Am. J. Clin. Nutr..

[36] Frank G.C. (1991). Taking a bite out of eating behavior: Food records and food recalls of children. J. Sch. Health.

[37] Department of Human Nutrition (2000). Diet Assessment Photos.

[38] Cole T.J., Flegal K.M., Nicholls D., Jackson A.A. (2007). Body mass index cut offs to define thinness in children and adolescents: International survey. BMJ.

[39] Cole T.J., Bellizzi M.C., Flegal K.M., Dietz W.H. (2000). Establishing a standard definition for child overweight and obesity worldwide: International survey. BMJ.

[40] Kuczmarski R.J., Ogden C.L., Grummer-Strawn L.M., Flegal K.M., Guo S.S., Wei R., Mei L.R., Curtin L.R., Roche A.F., Johnson C.L. (2000). CDC growth charts: United States. Adv. Data.

[41] Engle A., Lynn L.L., Koury K., Boyar A.P. (1990). Reproducibility and comparability of a computerized, self-administered food frequency questionnaire. Nutr. Cancer.

[42] Øverby N.C., Johannesen E., Jensen G., Skjaevesland A.K., Haugen M. (2014). Test-retest reliability and validity of a web-based food-frequency questionnaire for adolescents aged 13–14 to be used in the Norwegian Mother and Child Cohort Study (MoBa). Food Nutr. Res..

[43] Cade J.E., Burley V.J., Warm D.L., Thompson R.L., Margetts B.M. (2004). Food-frequency questionnaires: A review of their design, validation and utilisation. Nutr. Res. Rev..

[44] Cohen J. (1988). Statistical Power Analysis for the Behavioral Sciences.

[45] Cicchetti D.V. (1994). Guidelines, criteria, and rules of thumb for evaluating normed and standardized assessment instruments in psychology. Psychol. Assess..

[46] Wilson J.H. (2004). Essential Statistics.

[47] Andersen L.F., Bere E., Kolbjornsen N., Klepp K.I. (2004). Validity and reproducibility of self-reported intake of fruit and vegetable among 6th graders. Eur. J. Clin. Nutr..

[48] Vereecken C.A., Maes L. (2003). A Belgian study on the reliability and relative validity of the Health Behaviour in School-Aged Children food-frequency questionnaire. Public Health Nutr..

[49] Rockell J.E., Skidmore P.M.L., Parnell W.R., Wilson N. (2011). What children eat during afternoons and evenings: Is it important?. Public Health Nutr..

[50] Metcalf P.A., Scragg R.K.R., Sharpe S., Fitzgerald E.D.H., Schaaf D., Watts C. (2003). Short-term repeatability of a food frequency questionnaire in New Zealand children aged 1–14 years. Eur. J. Clin. Nutr..

[51] Del Pino D.L., Friedman R. (2011). Adaptation and validation of an FFQ for 6–10-year-old children. Public Health Nutr..

[52] Matos S.M.A., Prado M.S., Santos C.A.S.T., D’Innocenzo S., Assis A.M.O., Dourado L.S., Oliveira N.S., Rodrigues L.C., Barreto M.L. (2012). Validation of a food frequency questionnaire for children and adolescents aged 4 to 11 years living in Salvador, Bahia. Nutr. Hosp..

[53] Pampaloni B., Bartolini E., Barbieri M., Piscitelli P., Di Tanna G.L., Giolli L., Brandi M.L. (2013). Validation of a food-frequency questionnaire for the assessment of calcium intake in schoolchildren aged 9–10 years. Calcif. Tissue Int..

[54] Scagliusi F.B., Garcia M.T., Indiani A.L.C., Cardoso M.A. (2011). Relative validity of a food-frequency questionnaire developed to assess food intake of schoolchildren living in the Brazilian Western Amazon. Cad. Saúde Pública.

[55] Wilson A.M.R., Lewis R.D. (2004). Disagreement of energy and macronutrient intakes estimated from a food frequency questionnaire and 3-day diet record in girls 4 to 9 years of age. J. Am. Diet. Assoc..

[56] Haftenberger M., Heuer T., Heidemann C., Kube F., Krems C., Mensink G.B.M. (2010). Relative validation of a food frequency questionnaire for national health and nutrition monitoring. Nutr. J..

[57] Rodriguez G., Sjoberg A., Lissner L., Moreno L.A., Moreno L.A., Pigeto I., Ahrens W. (2011). Food patterns and nutrient intake in relation to childhood obesity. Epidemiology of Obesity in Children and Adolescents.

[58] Thompson F., Subar A., Coulston A., Boushey C., Ferruzzi M.G. (2008). Dietary assessment methodology. Nutrition in the Prevention and Treatment of Disease.

[59] Quigley R., Taylor R., Scragg R. (2007). Is Consuming Breakfast Important for Academic Performance, Maintaining A Healthy Body Weight, and Improving Nutrient Intake and Lifestyle Habits in Children.

[60] Block G., Thompson F.E., Hartman A.M., Larkin F.A., Guire K.E. (1992). Comparison of two dietary questionnaires validated against multiple dietary records collected during a 1-year period. J. Am. Diet. Assoc..

[61] Araujo M.C., Yokoo E.M., Pereira R.A. (2010). Validation and calibration of a semiquantitative food frequency questionnaire designed for adolescents. J. Am. Diet. Assoc..

[62] Truthmann J., Mensink G.B.M., Richter A. (2011). Relative validation of the KiGGS Food Frequency Questionnaire among adolescents in Germany. Nutr. J..

[63] Kolodziejczyk J.K., Merchant G., Norman G.J. (2012). Reliability and validity of child/adolescent food frequency questionnaires that assess foods and/or food groups. J. Pediatr. Gastroenterol. Nutr..

